# Circulating Inflammatory Markers Are Inversely Associated with Heart Rate Variability Measures in Type 1 Diabetes

**DOI:** 10.1155/2020/3590389

**Published:** 2020-08-18

**Authors:** Anne-Marie L. Wegeberg, Tina Okdahl, Tina Fløyel, Christina Brock, Niels Ejskjaer, Sam Riahi, Flemming Pociot, Joachim Størling, Birgitte Brock

**Affiliations:** ^1^Mech-Sense, Department of Gastroenterology and Hepatology, Aalborg University Hospital, Aalborg, Denmark; ^2^Clinical Institute, Aalborg University, Aalborg, Denmark; ^3^Steno Diabetes Center Copenhagen, Gentofte, Denmark; ^4^Steno Diabetes Center North Denmark, Aalborg University Hospital, Aalborg, Denmark; ^5^Department of Endocrinology, Aalborg University Hospital, Aalborg, Denmark; ^6^Department of Cardiology, Aalborg University Hospital, Denmark; ^7^Faculty of Health and Medical Sciences, University of Copenhagen, Copenhagen, Denmark; ^8^Department of Biomedical Sciences, University of Copenhagen, Copenhagen, Denmark

## Abstract

**Introduction:**

A neuroimmune communication exists, and compelling evidence suggests that diabetic neuropathy and systemic inflammation are linked. Our aims were (1) to investigate biomarkers of the ongoing inflammation processes including cytokines, adhesion molecules, and chemokines and (2) to associate the findings with cardiovascular autonomic neuropathy in type 1 diabetes by measuring heart rate variability and cardiac vagal tone.

**Materials and Methods:**

We included 104 adults with type 1 diabetes. Heart rate variability, time domain, and frequency domains were calculated from a 24-hour Holter electrocardiogram, while cardiac vagal tone was determined from a 5-minute electrocardiogram. Cytokines (interleukin- (IL-) 1*α*, IL-4, IL-12p70, IL-13, IL-17, and tumor necrosis factor- (TNF-) *α*), adhesion molecules (E-selectin, P-selectin, and intercellular adhesion molecule- (ICAM-) 1), and chemokines (chemokine (C-C motif) ligand (CCL)2, CCL3, CCL4, and C-X-C motif chemokine (CXCL)10) were assessed using a Luminex multiplexing technology. Associations between concentrations of inflammatory biomarkers and continuous variables of heart rate variability and cardiac vagal tone were estimated using multivariable linear regression adjusting for age, sex, disease duration, and smoking.

**Results:**

Participants with the presence of cardiovascular autonomic neuropathy had higher systemic levels of IL-1*α*, IL-4, CCL2, and E-selectin than those without cardiovascular autonomic neuropathy. IL-1*α*, IL-4, IL-12, TNF-*α*, and E-selectin were inversely associated with both sympathetic and parasympathetic heart rate variability measures (*p* > 0.01). *Discussion*. Our results show that several pro- and anti-inflammatory factors, believed to be involved in the progression of diabetic polyneuropathy, are associated with cardiovascular autonomic neuropathy, suggesting that these factors may also contribute to the pathogenesis of cardiovascular autonomic neuropathy. Our findings emphasize the importance of the neuroimmune regulatory system in the pathogenesis of neuropathy in type 1 diabetes.

## 1. Introduction

There exists a tight integration between the immune and nervous systems, the so-called inflammatory reflex, capable of influencing both branches in response to inflammatory and infectious agitation of homeostasis. Essentially, the autonomic nervous system is implicated in a bidirectional inflammatory reflex with the vagal nerve being the main neuronal substrate of an immunoregulatory role, providing a fast and subconscious anti-inflammatory response [1, 2]. Through activation of the inflammatory reflex, cytokine release actuates the brain or the vagal afferents directly. Afferent information is relayed to the brainstem and hypothalamus, which induces a cholinergic anti-inflammatory reflex mediated by acetylcholine in the vagal efferent branches. Consequently, a deactivation of macrophages and subsequently inhibition of the synthesis of primarily proinflammatory cytokines delineate the neuroimmune communication [1–3].

Cardiovascular autonomic neuropathy (CAN) is relatively common in diabetes, and its presence is associated with increased morbidity and mortality but has not traditionally been characterized by an inflammatory component [1, 4]. Due to its length, the vagal nerve is particularly vulnerable to long-term hyperglycaemia and is therefore severely affected in CAN. Consequently, the inflammatory reflex may have an implication in the pathogenesis of CAN and other microvascular complications in diabetes [5]. Heart rate is the base component for standardized heart rate variability (HRV) and cardiac vagal tone (CVT). HRV measures the fluctuations between subsequent heartbeats, indexing neurocardiac function, which can be quantified by the validated time and frequency domains [6], while CVT is a validated measure of vagal efferent activity from the brainstem to the heart [7, 8]. In diabetes, reduced HRV are highly sensitive for the detection of CAN and precede clinical symptoms [1, 9].

The link between the inflammatory reflex and HRV is further substantiated in healthy individuals, as endotoxin-induced subclinical inflammation reduces HRV, while increased parasympathetic vagal activity decreases cytokine production [3, 10]. Similar findings have been reported in type 2 diabetes, although these are highly associated with the presence of adipose inflammation [4, 11–13]. Systemic low-grade inflammation has been linked to CAN in long-term type 1 diabetes [5, 14]; however, in both adolescent and recent onset diabetes, the inflammatory levels are not associated with HRV, suggesting that a neurodegenerative inflammation emerges years after diabetes onset [11, 13]. Hence, inflammatory contributions to the pathogenesis of CAN likely differs between type 1 and type 2 diabetes. Furthermore, exploration of possible inflammatory mediators has been limited by the fact that most studies only investigate acute C-reactive protein, interleukin- (IL-) 6, tumour necrosis factor- (TNF-) *α*, or TNF-*α* receptors 1 and 2 [5, 14]. However, implementation of multiplexing technologies has broadened the spectrum of available biomarkers of chronic low-grade systemic inflammation.

In this study, we hypothesised that increased low-grade inflammation in adults with long-term type 1 diabetes would be associated with altered neurocardiac function. Therefore, we aimed to investigate a broad profile of inflammatory cytokines, adhesion molecules, and chemokines and their association with cardiovascular autonomic function assessed with HRV and CVT.

## 2. Materials and Methods

### 2.1. Study Population

The study population consisted of 104 adults with type 1 diabetes recruited from the Department of Endocrinology at Aalborg University Hospital, Denmark. Fifty-one had severe distal symmetrical polyneuropathy verified by abnormal nerve conduction testing, previously described by Brock et al. [15], while the remaining 53 showed no clinical signs and symptoms of distal symmetrical polyneuropathy. Eligible participants were ≥18 years old, of Northern European decent with verified type 1 diabetes mellitus for more than one year (HbA1c ≥ 6.5%). Participants were on stable hyperglycemic medication, including long-acting and fast-acting insulin or insulin pumps with dose adjustment according to the regimens minimum 1 month prior to study entry. Exclusion criteria have previously been described in detail [16]. The study was conducted in accordance with the Declaration of Helsinki, local regulations, and International Conference on Harmonization Good Clinical Practice guidelines. The protocol, amendments, and the informed consent form were approved by The North Denmark Region Committee on Health Research Ethics, Denmark (N-20130077 and N-20170045). All subjects gave their informed consent.

### 2.2. Heart Rate Variability

Twenty-four-hour electrocardiogram was obtained using either a Holter monitor (Lifecard CF; Del Mar Reynolds, Spacelabs Healthcare, Snoqualmie, WA, USA) or the extended patch-type Holter monitor ePatch electrocardiographic Recorder (BioTelemetry Technology ApS, Hørsholm, Denmark). The monitor was mounted in the morning, and assessment of HRV parameters was computed throughout the 24-hour recording period during which normal daily activities and routines were allowed. Data was analyzed using Pathfinder (Software revision B code; Spacelabs Healthcare) and Cardiscope™ (SmartMedical, Gloucestershire, United Kingdom), respectively. Time-derived measures (standard deviation of all normal RR (SDNN), standard deviation of the averages of RR (SDANN), mean standard deviation of the averages of RR for each 5-minute interval (SDNNi), and root mean square of the successive differences (RMSSD)) and frequency domain measures (very low frequency (VLF), low frequency (LF), and high frequency (HF)) were analyzed and interpreted according to international guidelines [9].

### 2.3. Cardiac Vagal Tone

Cardiac vagal tone was obtained using a simple 3-lead electrocardiography (eMotion Faros 180 device (Bittium, Oulu, Finland)), with electrodes (Ambu Blue Sensor P, Ballerup, Denmark) placed on the right and left subclavicular areas and at the cardiac apex. Participants were asked to refrain from coffee two hours prior to testing, and the test was conducted following a 5-minute relaxed period. CVT was computed by the ProBioMetrics online app version 1.0 (ProBioMetrics, Kent, United Kingdom). Recording artifacts were defined as a sudden change in two succeeding heart beats exceeding 15 beats per minute in variation (e.g., coughing or movement), and consequently files were cleaned by removing five heartbeats before and after, in order to derive the true CVT. The file was discarded if the number of edited heartbeats exceeded 20%. Stratification of participants into CAN–no CAN was based on a CVT cut-off value of 3.18

### 2.4. Serum Concentrations of Inflammation Markers

Blood samples were drawn from the cubital vein of fasting participants on the day of examination and centrifuged at 2500 rpm/10 minutes, and serum was stored in -80°C freezer until analysis were performed. Serum concentrations of cytokines (interleukin- (IL-) 1*α*, IL-1*β*, IL-4, IL-6, IL-8, IL-10, IL-12p70, IL-13, IL-17A, interferon- (IFN-) *α*, IFN-*γ*, tumor necrosis factor- (TNF-) *α* and granulocyte-macrophage colony-stimulating factor (GM-CSF)), adhesion molecules (E-selectin, P-selectin, and intercellular adhesion molecule (ICAM)-1), and chemokines (chemokine (C-C motif) ligand (CCL)2, CCL3, CCL4, and C-X-C motif chemokine (CXCL)10) were analyzed in duplicates by Luminex multiplexing technology using the Inflammation 20-Plex Human ProcartaPlex™ Panel (Invitrogen, Thermo Fisher Scientific, Waltham, MA, USA) and a MAGPIX instrument (Luminex, Austin, TX, USA), according to the manufacturer's protocol. If a sample exceeded three standard deviations from the group-mean, it was considered an outlier and subsequently removed before analysis. In addition, if a sample was below the detection level, the sample value was transformed by dividing the detection level value with the square root of 2 [16]. Readings with a value of zero or with a coefficient of variation (CV) above 20% between duplicates were excluded from the dataset.

### 2.5. Statistics

Data is presented as mean ± SD, median (25^th^-75^th^ percentiles), or number (%), dependent on data type and distribution. We tested for differences between groups (CAN–no CAN) with Student's *t*-test, the Mann–Whitney *U*-test, and the chi-squared test. Associations between concentrations of inflammatory markers and continuous variables of HRV and CVT were estimated using multivariable linear regression models (separate models for the associations of each of the inflammation markers with each of the eight variables of cardiovascular autonomic dysfunction) with increasing complexity (0: unadjusted; 1: adjusted for age and sex; 2: adjusted for age, sex, and disease duration; and 3: adjusted for age, sex, disease duration, and smoking).

All statistical analyses were performed using proprietary software (Stata version 15.0, StataCorp, Texas, USA). Statistical significance was inferred at a two-tailed *p* value < 0.01. Analyses were not adjusted for multiple comparisons and should therefore be considered exploratory.

## 3. Results

All participants completed the study with no adverse events; however, one participant was excluded from the analysis due to daily consumption of Methotrexate. We successfully measured six cytokines, three adhesion molecules, and four chemokines. The following inflammatory markers could not be detected or were only detected in very few of the samples: IL-1*β*, IL-6, IL-8, IL-10, IFN-*α*, IFN-*γ*, and GM-CSF.

### 3.1. Study Population

Demographic characteristics of the study participants based on the presence of CAN can be found in Table 1. No differences were seen between the groups with respect to sex, BMI, smoking status, HbA1c, diastolic blood pressure, cholesterol levels, or insulin use. Participants with the presence of CAN (e.g., lower CVT) were predominantly males (69% vs 49%), who were older (51 vs. 41 years), had longer disease duration (32 vs 22 years), and higher blood pressure (systolic 141 vs 132 and diastolic 78 vs 74) and were more likely to have distal symmetrical polyneuropathy (69% vs 29%) in comparison to the group with no CAN (Table 1). Furthermore, they had decreased time and frequency domain parameters of HRV, namely, SDNNi (44 vs 57), RMSSD (20 vs 30), VLF (1091 vs 1861), LF (375 vs 968), and HF (101 vs. 268).

### 3.2. Systemic Level of inflammatory Markers

**Figure 1:**
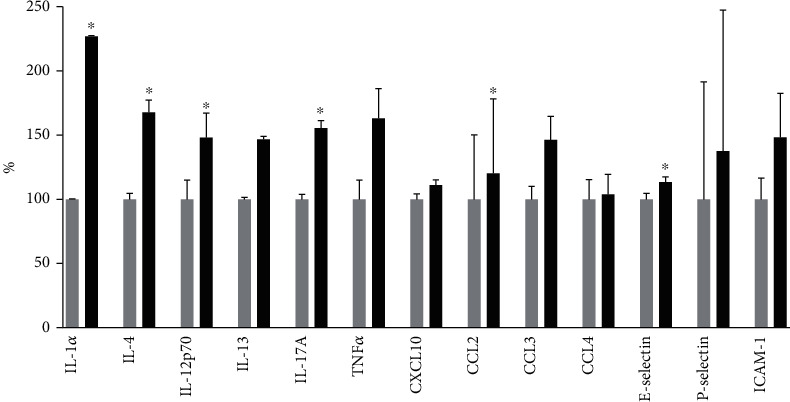
Increase in inflammatory markers in participants with cardiovascular autonomic neuropathy measured with cardiac vagal tone below 3.18 (black columns) is compared to those without (cardiac vagal tone above 3.18) (grey columns). The difference is in percentage compared to those without cardiovascular autonomic neuropathy. ^∗^*p* < 0.05 when comparing participants with and without CAN.

Participants with the presence of CAN had significantly higher systemic levels of IL-1*α*, IL-4, CCL2, and E-selectin, than those with no CAN, whereas the systemic levels of IL-1*β*, IL-12p70, IL-13, IL-17A, TNF-*α*, CXCL10, CCL3, CCL4, P-selectin, and ICAM-1, though all are numerically higher, did not statically differ (Figure 1).

### 3.3. Cytokines and Cardiovascular Autonomic Dysfunction

Independent of the CAN status, initial unadjusted linear regression analysis indicated 35 significant inverse associations between the concentrations of inflammatory cytokines and parameters of cardiovascular autonomic dysfunction, with statistically significant associations for all cytokines (Table 2). However, after adjusting for age and sex (and subsequently disease duration and smoking), only 15 inverse associations remained, see Table 2. After adjustments, IL-1*α* was associated with SDNNI and RMSSD; IL-4 was associated with SDNNi, RMSSD, and LF; IL-12p70 was associated with SDNN, SDNNi, RMSSD, VLF, and LF; and TNF-*α* was associated with SDNNi, RMSSD, VLF, LF, and HF. None of the associations for IL-13 and IL-17A remained after adjustments, neither did the initial association for CVT. Sex appears to be a contributing factor to the relationship between HRV parameters and IL-4 or TNF-*α*, while age was a contributing factor to the relationship between TNF-*α* and LF, and disease duration between TNF-*α* and HF.

### 3.4. Adhesion Molecules, Chemokines, and Cardiovascular Autonomic Dysfunction

Independent of CAN status, initial unadjusted analysis indicated 13 significant inverse associations between adhesion molecules or chemokines and parameters of cardiovascular autonomic dysfunction (Table 3). After adjusting for age and sex (and subsequently disease duration and smoking), only six associations with E-selectin persisted, indicating the relevance of this endothelial marker, see Table 3. E-selectin was inversely associated with SDNN, SDANN, SDNNi, RMSSD, VLF, and LF. Smoking appears to be a contributing factor to the relationship between E-selectin and RMSSD.

## 4. Discussion

In adults with type 1 diabetes, we found that the inflammatory cytokines IL-1*α*, IL-4, IL-12p70, TNF-*α*, and the adhesion molecule E-selectin were inversely associated with 24-hour-derived HRV parameters after adjustments for age, sex, disease duration, and smoking. These associations were not found for the 5-minute recordings of CVT. Additionally, as compared to individuals without signs of CAN, participants with the presence of CAN had higher systemic levels of IL-1*α*, IL-4, CCL2, and E-selectin.

### 4.1. Both Pro- and Anti-inflammatory Cytokines Associate with Heart Rate Variability

The existing relationship between the presence of diabetic neuropathy and proinflammatory cytokines, e.g., TNF-*α*, has been extensively studied. However, evidence of associations between low-grade inflammation and CAN is sparse [5, 17]. In animals, proinflammatory cytokines IL-1*α*, IL-1*β*, and TNF-*α* are known contributors to the pathogenesis of neuropathy [18]. It is likely that similar mechanisms may be involved in the development of CAN in humans.

TNF-*α* is a predominant mediator of proinflammatory processes, as it activates immune cells to release other proinflammatory cytokines. It is primarily produced by activated macrophages but can also be synthesized by CNS neurons [2]. It has previously been shown that systemically increased TNF-*α* levels are present in type 1 diabetes with increased blood pressure or cardiovascular disease [19, 20]. Complementarily,, we found participants with CAN to have increased levels of TNF-*α* and a persistent, strong inverse association between higher levels of TNF-*α* and decreased 24-hour HRV measures, e.g., SDNNi, RMSSD, VLF, LF, and HF, confirming previous findings. The 24-hour derived measures of HRV were expectedly superior and more robust to detect neurocardiac changes than the 5-minute recording of CVT, which is a screening measure of putative vagal parasympathetic tone.

Chronic hyperglycemia increases TNF-*α*, interleukins, and adhesion molecules due to containment of macrophages in a proinflammatory state [21, 22]. Both TNF-*α* and IL-1*α*/*β* are neuropoietic cytokines and are as such involved in the pathogenesis of nerve damage, including phagocytosis and demyelination. Individuals with type 1 diabetes have decreased neuronal regenerative potential, e.g., in comparison to healthy and type 2 diabetes, and this toxic combination may reflect the strong association between HRV measures and proinflammatory cytokines [23]. However, to our knowledge, no study has investigated IL-1*α* in adults with long-term diabetes. IL-1*α* is arranged in vesicles within the cell membrane in its mature form, seldom appearing in systemic circulation and detectable levels may therefore indicate cell death [24]. We found that IL-1*α* was inversely associated with SDNNi and RMSSD. IL-1*α* exerts its function essentially similar to IL-1*β* [24], and the latter has previously been inversely associated with changes in HF and RMSSD [18], suggesting a link to CAN.

A novel finding was that the anti-inflammatory cytokines IL-4 and IL-12p70 were also inversely associated with SDNNi, RMSSD, and LF, and IL-12p70 furthermore with SDNN and VLF. Though IL-13 structurally and partly functionally is similar to IL-4, this cytokine was not associated with HRV measures after adjusting for age and gender, emphasizing the complexity of the immunoregulatory system in diabetes.

### 4.2. Increased E-Selectin Suggests Epithelial Dysfunction as Part of Cardiovascular Dysfunction

Adhesion molecules E-selectin, P-selectin, and ICAM-1 are known biomarkers of vascular inflammation, expressed by arterial endothelial cells as an essential part of the chain reaction in differentiating macrophages from monocytes [13, 25]. E-selectin and P-selectin have been shown to be increased during the development of cardiovascular disease [25]. We found strong and robust associations between E-selectin and HRV parameters, suggesting an impact of epithelial dysfunction as part of the microvascular complication leading to CAN in diabetes.

Along the same line, ICAM-1 is a known predictor of cardiovascular risk [25, 26]. However, Herder et al. found no association between ICAM and HRV measures in recent maturity-onset type 1 diabetes [11]. Our results complement these findings in adults with long-term type 1 diabetes. This lack of association could possibly reflect general applications of an intensive treatment regime as standard, as the Diabetes Control and Complications Trial found levels of ICAM-1 increased over a three-year period during conventional treatment, in contrast to a decline during intensive insulin treatment [26].

### 4.3. Chemotactic Cytokines Are Not Associated with Cardiovascular Dysfunction

Chemokines play essential roles in local inflammation, by attracting immune cells to the site of injury or inflammation [27–29]. Previous studies have reported CCL2 levels to be independent predictors of cardiovascular events [28, 29], and coherently, we found CCL2 to be increased in participants with CAN. Contradictory, Guan et al. found no associations between CCL2 levels and autonomic neuropathy in type 1 diabetes; however, they suggested CCL2 to have a role in diabetes complications [27]. The overall lack of association between chemokines and autonomic parameters may be attributed to the administration of insulin in type 1 diabetes, which is known to have anti-inflammatory effects, thereby possibly reducing the expression of chemokines [29]. Taken together, our results suggest that the chemokine levels remain unaffected in the presence of CAN, questioning whether they play a role in CAN pathogenesis.

### 4.4. Treatment of Cardiovascular Dysfunction May Impact the Inflammatory Profile

On account of the evident intertwining vagal dysfunction and inflammation, treatment of either axis may induce an effect on both. Vagal stimulation through neuromodulation has been suggested as a potential treatment. Direct vagal nerve stimulation is known to reduce the proinflammatory response of cytokines including TNF-*α* and chemokines effectively in healthy individuals and in rheumatoid arthritis [23, 24]. Furthermore, beta-blockers have been shown to influence both the heart rate and inflammation levels [1]. In people with type 1 diabetes and decreased HRV parameters, intervention for four weeks with beta-blocker (atenolol) not only increased HRV measures but also decreased the levels of the inflammatory marker C-reactive protein [14]. It is therefore plausible that future mechanism-targeting treatments, e.g., lowering the systemic level of proinflammatory cytokines, adhesion molecules, and chemokines ultimately may lead to prevention or treatment of the neuroinflammatory component in subclinical stages of CAN. If successful, this would function as an example of how mechanistic principles can be translated into clinical practice similar to those applied in the cardiovascular and nephrological clinics for the benefit of future patients.

### 4.5. Limitations

This study has a number of limitations. First, the causation cannot be attributed due to the cross-sectional design of the study. Furthermore, we were limited by the measure of the inflammatory markers at a single time point, which may not accurately reflect average long-term levels. However, as serum samples and electrocardiograms were obtained within hours of each other, the results should at least reflect the acute interaction. Second, although 24-hour HRV is the gold standard within diabetes research and is known to be reproducible and stable over time, it is also influenced by cardiac reactions, changing workloads, and circadian processes [6, 9]. We used two validated commercially available software programs to analyse HRV parameters, and concerns have been raised regarding interpretation of the spectral indices as these provide averages of the modulations attributable to especially the LF and HF components [9]. Third, although multiplex systems enable acquisition of larger quantitative data in a cost-efficient manner, the risk of cross-reactions and the dynamic range of concentrations are some of the limitations to this method that may also hamper direct comparisons with other studies. In the present study, 7 out of 20 inflammation markers had too low detection levels to be assessed. Fourth, we used a CVT cut-off for description of CAN and not the gold standard cardiovascular autonomic reflex testing, which could result in an underestimation of the observed differences between the two investigated diabetic groups. Finally, multiple testing may have resulted in an inflation of the type 1 error rate. However, as we have lowered the inferred *p* value, we believe that our analyses are informative exploratory, possibly providing a basis for further research into the pathogenesis of CAN.

## 5. Conclusions

In conclusion, the obtained results support our hypothesis that circulating inflammatory factors are associated with putative sympathetic and parasympathetic HRV measures, independent of age, sex, disease duration, and smoking. This suggests that inflammatory factors, believed to be involved in nerve damage and epithelial dysfunction, may contribute to the pathogenesis of CAN. Furthermore, the results emphasize the complexity of the immunoregulatory system in type 1 diabetes.

## Figures and Tables

**Table 1:** Demographic and clinical characteristics among groups.

	CAN (CVT ≤ 3.18)	%CAN (CVT > 3.18)	*p* value
Age (years)	51 ± 10	41 ± 14	<0.01
Sex (males)	69%	49%	0.09
Body mass index (kg/m^2^)	27.1 (25.2–30.5)	25.1 (23.3–27.8)	0.02
Current smokers	17%	27%	0.27
Disease duration (years)	32 ± 11	22 ± 11	<0.01
Haemoglobin A1c (mmol/mol)	66 (57–75)	60 (53–69)	0.03
Systolic blood pressure (mmHg)	141 ± 15	132 ± 15	<0.01
Diastolic blood pressure (mmHg)	78 ± 9	74 ± 8	0.06
Heart rate (beats/min)	76 (72–80)	69 (64–75)	<0.01
Cholesterol (mmol/L)	4.2 (3.7–4.7)	4.4 (3.8–4.9)	0.42
Long acting insulin dose (IU)	26 (19–38)	24 (18–31)	0.31
Short acting insulin dose (IU)	27 (20–36)	22 (15–28)	0.11
Insulin pump dose (IU)	21.2 (19.9–49.6)	42.4 (32.0–50.0)	0.11
Distal symmetrical polyneuropathy (%)	69%	29%	<0.01
SDNN	112 (99–141)	136 (112-167)	0.02
SDANN	103 (88–131)	118 (99–145)	0.04
SDNNi	44 ± 19	57 ± 19	<0.01
RMSSD	20 (13–27)	30 (24–40)	<0.01
VLF	1091 (590-1479)	1861 (1184-2907)	<0.01
LF	375 (107–576)	968 (509-1580)	<0.01
HF	101 (38–197)	268 (141–615)	<0.01
Interleukin-1*α* (pg/mL)	1.0 (0.4–1.2)	0.5 (0.4–0.8)	0.01
Interleukin-4 (pg/mL)	15.9 (12.4–21.6)	7.4 (7.4–12.7)	<0.01
Interleukin-12p70 (pg/mL)	46.1 (38.9–65.7)	39.2 (33.2–48.5)	0.02
Interleukin-13 (pg/mL)	3.1 (2.1–4.4)	2.1 (2.1–3.7)	0.12
Interleukin-17A (pg/mL)	10.0 (7.5–15.2)	8.2 (5.9–10.9)	0.02
Tumor necrosis factor-*α* (pg/mL)	36.9 (22.9–54.5)	27.7 (19.7–38.5)	0.07
C-X-C motif chemokine 10 (pg/mL)	12.4 ± 4.1	11.1 ± 4.0	0.17
C-C motif chemokine ligand 2 (pg/mL)	88.4 (65.2–136.8)	64.2 (41.2–102.2)	<0.01
C-C motif chemokine ligand 3 (pg/mL)	14.5 (9.4–22.6)	13.0 (6.4–25.4)	0.54
C-C motif chemokine ligand 4 (pg/mL)	30.2 ± 16.6	31.6 ± 14.1	0.70
E-selectin (ng/mL)	16.8 ± 4.6	14.7 ± 4.4	<0.01
P-selectin (ng/mL)	180.8 (117.0 – 245.9)	143.4 (80.3 – 219.3)	0.06
Intercellular adhesion molecule 1 (ng/mL)	44.6 (26.4–57.4)	39.7 (27.7–57.6)	0.60

Based on normative distribution, data is presented as mean ± SD or median (25th-75th percentiles) for continuous variables and number (%) for binary variables. *p* values between the groups (CAN–No CAN) were based on Student's *t*-test, the Mann–Whitney *U*-test, and the chi-squared test. SDNN: standard deviation of all normal RR; SDANN: standard deviation of the averages of RR: SDNNi: mean standard deviation of the averages of RR for each 5-minute interval; RMSSD: root mean square of the successive differences; VLF: very low frequency; LF: low frequency; HF: high frequency; UI: international units.

**Table 2:** Regression analyses of cytokines and neurocardiac.

		Unadjusted		Adjusted 1		Adjusted 2		Adjusted 3	
		*ρ*	*p*	*R* ^2^	*p*	*R* ^2^	*p*	*R* ^2^	*p*
IL-1*α*	CVT	**-0.41**	**<0.001**	0.21	0.018	0.24	0.039	0.24	0.052
	SDNN	**-0.34**	**0.003**	0.21	0.030^†^	0.24	0.026	0.25	0.028
	SDANN	**-0.31**	**0.006**	0.20	0.041^†^	0.24	0.033	0.24	0.035
	SDNNi	**-0.45**	**<0.001**	**0.26**	**0.002**	**0.28**	**0.004**	**0.28**	**0.004**
	RMSSD	**-0.43**	**<0.001**	**0.25**	**0.003** ^†^	**0.28**	**0.004**	**0.28**	**0.005**
	VLF	**-0.37**	**0.001**	0.21	0.046^†^	0.24	0.043	0.24	0.048
	LF	**-0.44**	**<0.001**	0.23	0.015	0.25	0.017	0.26	0.018
	HF	**-0.36**	**0.002**	0.21	0.042^†^	0.24	0.040	0.24	0.048

IL-4	CVT	**-0.36**	**0.002**	0.15	0.028	0.17	0.054	0.18	0.100
	SDNN	**-0.30**	**0.006**	0.16	0.033^‡^	0.19	0.032	0.19	0.035^‡^
	SDANN	-0.26	0.016	0.15	0.070^‡^	0.18	0.066	0.18	0.069^‡^
	SDNNi	**-0.36**	**<0.001**	**0.20**	**0.005** ^‡^	**0.22**	**0.008**	**0.22**	**0.009** ^‡^
	RMSSD	**-0.40**	**<0.001**	**0.22**	**0.002** ^‡^	**0.23**	**0.003**	**0.23**	**0.004** ^‡^
	VLF	**-0.31**	**0.005**	0.18	0.024^‡^	0.20	0.025	0.20	0.030^‡^
	LF	**-0.33**	**0.002**	**0.20**	**0.007** ^‡^	**0.22**	**0.009**	**0.22**	**0.010** ^‡^
	HF	-0.26	0.022	0.15	0.089^‡^	0.17	0.103	0.18	0.129^‡^

IL-12p70	CVT	-0.28	0.013	0.09	0.075	0.11	0.128	0.12	0.097
	SDNN	**-0.35**	**0.001**	**0.15**	**0.006**	**0.19**	**0.005**	**0.19**	**0.006**
	SDANN	**-0.30**	**0.006**	0.13	0.018	0.16	0.017	0.17	0.019
	SDNNi	**-0.44**	**<0.001**	**0.21**	**0.000**	**0.23**	**0.000**	**0.24**	**0.001**
	RMSSD	**-0.44**	**<0.001**	**0.21**	**0.000**	**0.23**	**0.000**	**0.24**	**0.001**
	VLF	**-0.37**	**<0.001**	**0.17**	**0.003**	**0.20**	**0.003**	**0.20**	**0.003**
	LF	**-0.41**	**<0.001**	**0.20**	**0.001**	**0.23**	**0.001**	**0.23**	**0.001**
	HF	**-0.33**	**0.003**	0.14	0.016	0.17	0.012	0.18	0.012

IL-13	CVT	-0.24	0.042	0.13	0.272^‡^	0.14	0.358	0.15	0.295^‡^
	SDNN	-0.23	0.037	0.14	0.214^‡^	0.16	0.206	0.17	0.193^‡^
	SDANN	-0.18	0.120	0.13	0.425^‡^	0.15	0.415	0.16	0.406^‡^
	SDNNi	**-0.31**	**0.005**	0.18	0.034^‡^	0.19	0.045	0.20	0.043^‡^
	RMSSD	**-0.33**	**0.002**	0.19	0.013^‡^	0.15	0.017	0.22	0.013^‡^
	VLF	-0.24	0.032	0.15	0.132^‡^	0.20	0.141	0.18	0.132^‡^
	LF	**-0.30**	**0.006**	0.19	0.016^‡^	0.20	0.020	0.21	0.019^‡^
	HF	-0.24	0.032	0.16	0.093^‡^	0.17	0.089	0.19	0.070^‡^

IL-17A	CVT	**-0.32**	**0.003**	0.13	0.040	0.16	0.077	0.16	0.109
	SDNN	-0.24	0.022	0.15	0.175^‡^	0.19	0.181	0.19	0.185^‡^
	SDANN	-0.21	0.046	0.14	0.255^‡^	0.18	0.256	0.18	0.259^‡^
	SDNNi	**-0.34**	**0.001**	0.19	0.012^‡^	0.22	0.022	0.22	0.022^‡^
	RMSSD	**-0.32**	**0.002**	0.19	0.021^‡^	0.21	0.033	0.21	0.034^‡^
	VLF	**-0.28**	**0.009**	0.17	0.074^‡^	0.20	0.080	0.20	0.083^‡^
	LF	**-0.30**	**0.005**	0.18	0.033^‡^	0.21	0.044	0.21	0.046^‡^
	HF	-0.20	0.069	0.15	0.306^‡^	0.18	0.302	0.18	0.314^‡^

TNF-*α*	CVT	-0.28	0.013	0.15	0.035^‡^	0.16	0.068	0.18	0.046^‡^
	SDNN	**-0.34**	**0.002**	0.19	0.011^‡^	0.23	0.011	0.24	0.013^‡§^
	SDANN	-0.27	0.015	0.16	0.049^‡^	0.20	0.051	0.21	0.060^‡§^
	SDNNi	**-0.43**	**<0.001**	**0.27**	**0.000** ^‡^	**0.29**	**0.000**	**0.31**	**0.000** ^‡^
	RMSSD	**-0.47**	**<0.001**	**0.29**	**0.000** ^‡^	**0.31**	**0.000**	**0.33**	**0.000** ^‡^
	VLF	**-0.35**	**0.001**	**0.24**	**0.002** ^‡^	**0.26**	**0.002**	**0.28**	**0.002** ^‡^
	LF	**-0.37**	**<0.001**	**0.26**	**0.000** ^‡^	**0.28**	**0.001**	**0.29**	**0.001** ^†‡^
	HF	**-0.36**	**<0.001**	**0.23**	**0.003** ^‡^	**0.26**	**0.002**	**0.27**	**0.003** ^‡§^

The table provides rho or *R*^2^ and corresponding *p* values from linear regression models. Bold print indicates statistically significant associations. Adjusted 1: adjusted for age and sex. Adjusted 2: adjusted for age, sex, and disease duration. Adjusted 3: adjusted for age, sex, disease duration, and smoking. Significant adjusting factors: ^†^age, ^‡^sex, ^§^disease duration. IL: interleukin; TNF: tumor necrosis factor; CVT: cardiac vagal tone; SDNN: standard deviation of all normal RR; SDANN: standard deviation of the averages of RR; SDNNi: mean standard deviation of the averages of RR for each 5-minute interval; RMSSD: root mean square of the successive differences; VLF: very low frequency; LF: low fre-quency; HF: high frequency.

**Table 3:** Regression analyses of chemokines, adhesion molecules, and neurocardiac measures.

		Unadjusted		Adjusted 1		Adjusted 2		Adjusted 3	
		*ρ*	*p*	*R* ^2^	*p*	*R* ^2^	*p*	*R* ^2^	*p*
CXCL10	CVT	-0.27	0.015	0.18	0.048^‡^	0.21	0.067^‡^	0.21	0.087^‡^
	SDNN	-0.18	0.114	0.20	0.316^†‡^	0.23	0.268^‡^	0.23	0.267^‡^
	SDANN	-0.15	0.187	0.20	0.379^†‡^	0.23	0.307^‡^	0.23	0.302^‡^
	SDNNi	**-0.29**	**0.008**	0.22	0.108^†‡^	0.24	0.125^‡^	0.25	0.124^‡^
	RMSSD	-0.15	0.172	0.20	0.676^†‡^	0.22	0.701^‡^	0.22	0.713^‡^
	VLF	**-0.29**	**0.009**	0.22	0.164^†‡^	0.24	0.138^‡^	0.24	0.142^‡^
	LF	-0.28	0.012	0.20	0.557^†‡^	0.22	0.521^‡^	0.22	0.523^‡^
	HF	-0.19	0.091	0.20	0.744^†‡^	0.22	0.690^‡^	0.22	0.717^‡^

CCL2	CVT	**-0.29**	**0.007**	0.12	0.165	0.13	0.210	0.14	0.141
	SDNN	-0.18	0.088	0.11	0.390^†^	0.11	0.403	0.14	0.393
	SDANN	-0.13	0.206	0.11	0.587^†^	0.11	0.594	0.13	0.602
	SDNNi	**-0.29**	**0.005**	0.14	0.072	0.14	0.085	0.16	0.085
	RMSSD	**-0.29**	**0.006**	0.14	0.069	0.14	0.079	0.16	0.061
	VLF	**-0.30**	**0.005**	0.14	0.074	0.14	0.079	0.16	0.068
	LF	**-0.30**	**0.004**	0.14	0.082	0.14	0.091	0.16	0.086
	HF	-0.22	0.043	0.11	0.318	0.12	0.324	0.14	0.247

CCL3	CVT	-0.18	0.099	0.04	0.196	0.04	0.260	0.10	0.534^¶^
	SDNN	-0.05	0.649	0.04	0.984	0.05	0.983	0.10	0.953^¶^
	SDANN	-0.02	0.849	0.04	0.826	0.05	0.846	0.10	0.847^¶^
	SDNNi	-0.17	0.112	0.05	0.231	0.07	0.298	0.11	0.290^¶^
	RMSSD	-0.19	0.069	0.06	0.154	0.07	0.185	0.11	0.234
	VLF	-0.13	0.227	0.04	0.417	0.06	0.445	0.10	0.490^¶^
	LF	-0.17	0.108	0.06	0.182	0.07	0.226	0.11	0.237^¶^
	HF	-0.16	0.128	0.05	0.220	0.07	0.213	0.11	0.302

CCL4	CVT	-0.26	0.034	0.08	0.025	0.08	0.029	0.08	0.042
	SDNN	-0.15	0.207	0.03	0.277	0.03	0.277	0.05	0.311
	SDANN	-0.14	0.221	0.03	0.281	0.03	0.282	0.05	0.310
	SDNNi	-0.20	0.095	0.04	0.160	0.04	0.164	0.06	0.174
	RMSSD	-0.08	0.485	0.01	0.666	0.02	0.669	0.03	0.733
	VLF	-0.10	0.381	0.02	0.618	0.02	0.620	0.04	0.720
	LF	-0.05	0.685	0.01	0.910	0.01	0.911	0.04	0.877
	HF	0.05	0.682	0.03	0.384	0.03	0.386	0.06	0.293

E-selectin	CVT	-0.20	0.072	0.04	0.231	0.05	0.319	0.07	0.234
	SDNN	**-0.43**	**<0.001**	**0.20**	**<0.001**	**0.21**	**<0.001**	**0.24**	**<0.001**
	SDANN	**-0.44**	**<0.001**	**0.20**	**<0.001**	**0.21**	**<0.001**	**0.25**	**<0.001**
	SDNNi	**-0.43**	**<0.001**	**0.21**	**<0.001**	**0.21**	**<0.001**	**0.24**	**<0.001**
	RMSSD	**-0.36**	**<0.001**	**0.14**	**0.001**	**0.15**	**0.002**	**0.19**	**0.001** ^¶^
	VLF	**-0.37**	**<0.001**	**0.16**	**0.001**	**0.17**	**0.001**	**0.20**	**0.001**
	LF	**-0.36**	**<0.001**	**0.17**	**0.001**	**0.17**	**0.001**	**0.20**	**0.001**
	HF	-0.24	0.027	0.08	0.044	0.09	0.043	0.13	0.026

P-selectin	CVT	-0.17	0.126	0.07	0.261	0.08	0.313	0.13	0.161
	SDNN	-0.08	0.480	0.06	0.712	0.08	0.721	0.11	0.813
	SDANN	-0.06	0.593	0.05	0.791^‡^	0.08	0.798	0.11	0.936
	SDNNi	-0.14	0.194	0.07	0.255^‡^	0.09	0.318	0.12	0.377
	RMSSD	-0.12	0.266	0.06	0.418	0.08	0.484	0.12	0.482
	VLF	-0.13	0.230	0.07	0.274	0.09	0.281	0.13	0.304
	LF	-0.13	0.262	0.07	0.232^‡^	0.09	0.272	0.13	0.306
	HF	-0.14	0.212	0.06	0.303	0.09	0.284	0.13	0.272

ICAM-1	CVT	-0.22	0.057	0.13	0.265^‡^	0.13	0.302^‡^	0.13	0.266^‡^
	SDNN	-0.12	0.313	0.12	0.729^‡^	0.12	0.740^‡^	0.13	0.786^‡^
	SDANN	-0.07	0.546	0.12	0.977^‡^	0.12	0.980^‡^	0.13	0.902^‡^
	SDNNi	-0.17	0.134	0.13	0.268^‡^	0.13	0.310^‡^	0.14	0.327^‡^
	RMSSD	-0.24	0.031	0.15	0.084^‡^	0.15	0.100^‡^	0.16	0.099^‡^
	VLF	-0.20	0.079	0.14	0.149^‡^	0.15	0.157^‡^	0.15	0.158^‡^
	LF	-0.15	0.184	0.13	0.218^‡^	0.14	0.248^‡^	0.15	0.258^‡^
	HF	-0.20	0.080	0.14	0.141^‡^	0.15	0.142^‡^	0.16	0.139^‡^

The table gives rho or *R*^2^ and corresponding *p* values from linear regression models. Bold print indicates statistically significant associations. Adjusted 1: adjusted for age and sex. Adjusted 2: adjusted for age, sex, and disease duration. Adjusted 3: adjusted for age, sex, disease duration, and smoking. Significant adjusting factors: ^†^age, ^‡^sex, ^§^disease duration, ^¶^smoking. CXCL: C-X-C motif chemokine; CCL: C-C motif chemokine ligand; ICAM: intercellular adhesion molecule; CVT: cardiac vagal tone; SDNN: standard deviation of all normal RR; SDANN: standard deviation of the averages of RR; SDNNi: mean standard deviation of the averages of RR for each 5-minute interval; RMSSD: root mean square of the successive differences; VLF: very low frequency; LF: low frequency; HF: high frequency.

## Data Availability

The datasets used and/or analyzed during the current study are available from the corresponding author on reasonable request. This manuscript has been preprinted at https://www.researchsquare.com/article/rs-29588/v1.
